# Discussing male sexual and reproductive health in the rheumatology outpatient clinic: a Q-methodology study

**DOI:** 10.1186/s41927-024-00441-3

**Published:** 2024-12-05

**Authors:** L. F. Perez-Garcia, E. Röder, H. Pastoor, A. C. Lozada-Navarro, I. Colunga-Pedraza, T. Vargas-Aguirre, J. van Exel, A. Vargas-Guerrero, R. J. E. M. Dolhain

**Affiliations:** 1 Department of Rheumatology, Erasmus MC, University Medical Center, Doctor Molewaterplein 40, Rotterdam, GD 3015 the Netherlands; 2 Department of Obstetrics and Gynecology, Division of Reproductive Endocrinology and Infertility, Erasmus MC, University Medical Center, Rotterdam, the Netherlands; 3 Hospital Faro del Mayab, Mérida, Mexico; 4 Centre for Rheumatology, University Hospital “Dr. José Eleuterio González”, Monterrey, Mexico; 5 School of Medicine and Health Sciences, TecSalud, Monterrey, Mexico; 6 Erasmus School of Health Policy & Management, Erasmus University Rotterdam, Rotterdam, the Netherlands; 7 Department of Rheumatology, National Institute of Cardiology “Ignacio Chávez”, Mexico City, Mexico

**Keywords:** Rheumatoid arthritis, Sexual health, Reproductive health, Qualitative research, Patient care team

## Abstract

**Objectives:**

Inflammatory arthritis (IA) has been associated with various problems related to male sexual and reproductive health (SRH). However, addressing these issues in the clinic remains a challenge. In this study, we aimed to describe the viewpoints of rheumatologists and male patients with IA regarding the aspects that influence their communication about SRH.

**Methods:**

Rheumatologists and adult men with IA were invited to participate. This study uses Q-methodology, a mixed methods approach to systematically study subjectivity. Participants ranked 32 aspects according to their degree of influence (least-most influence) in addressing SRH and were then interviewed. Factor analysis was used to identify common patterns in the rankings. These patterns were interpreted as the different viewpoints of rheumatologists and male patients, supported by the qualitative data from the interviews. To obtain more generalizable results, the study was conducted in two countries with different socio-cultural backgrounds and healthcare systems, The Netherlands and Mexico.

**Results:**

30 rheumatologists and 30 men with IA were included in each country. The analysis revealed three viewpoints in each group. Rheumatologists are more likely to be influenced by aspects such as the patient’s desire to become a father or the patients’ (young) age, but patients by a much more diverse pool of aspects, such as potential side effects of medication on their sexual function.

**Conclusions:**

This study identified different viewpoints on the aspects that influence discussing SRH between rheumatologists and male patients, and important differences in viewpoints between both groups. Further research is needed to reach consensus on how and when rheumatologists and male patients should discuss SRH.

**Supplementary Information:**

The online version contains supplementary material available at 10.1186/s41927-024-00441-3.

## Key points

• For both groups, having an active wish to become a father can be considered the “automatic trigger” to discuss male sexual and reproductive health. Remarkably, while rheumatologists predominantly prioritize this aspect, patients—especially those without an active wish to become fathers—consider a broader spectrum of factors, emphasizing the need for a comprehensive understanding of patient concerns beyond reproductive issues.

• While patients highly value receiving information from rheumatologists about this topic, the expectation for rheumatologists to initiate these conversations, particularly regarding sexual health, suggests a potential hesitancy on the part of patients. Understanding and addressing this hesitancy is crucial in establishing effective communication practices in the context of sexual and reproductive health.

• Patients should be informed about the potential impact of sexual and reproductive health of inflammatory arthritis early during the course of their disease and provided with “facilitators” to discuss this topic throughout the course of their disease.

• Further research is required to establish consensus on how and when discussions about sexual and reproductive health should take place between rheumatologists and male patients diagnosed with inflammatory arthritis.

**Supplementary Information:**

The online version contains supplementary material available at 10.1186/s41927-024-00441-3.

## Introduction

“Sexual and reproductive health (SRH) is a state of complete physical, mental and social well-being and not merely the absence of disease or infirmity, in all matters relating to the reproductive system and to its functions and processes”. It also includes sexuality, “the purpose of which is the enhancement of life and personal relations, and not merely counselling and care related to reproduction and sexually transmitted diseases” [1].

It is estimated that between 36 and 70% of female and male patients diagnosed with inflammatory arthritis (IA) experience some form of impaired SRH and most of them do not discuss these problems with their rheumatologists [2–7].

More than 60% of men from the general population consider SRH as an important contributor to their quality of life [8] and more than 80% of patients deemed that a SRH history should be an integral part of medical consultations [9]. Correspondingly, it is now advised that SRH should be part of the standard clinicians’ assessment [10, 11]. Nonetheless, in Rheumatology, this topic is rarely addressed with male patients [12, 13].

While erectile dysfunction (ED) is a common issue in men with IA, it is not the only form of sexual dysfunction (SD) [14]. SD can also include decreased libido, premature ejaculation and anorgasmia, which can result from not only biological factors but also psychological or social factors [15]. Chronic pain, fatigue, anxiety, depression, and relationship issues often intersect with these factors contributing to SD [16]. Furthermore, it is estimated that 25% of all ED cases are related to medication use [17]. Immunosuppressive drugs used for the treatment of IA have been associated with ED [18, 19]. Men experiencing symptoms from severe ED are often reluctant to disclose their symptoms to their health care professionals (HCPs) [20] and because HCPs rarely address this topic with their patients, the actual frequency of medication-induced ED can be higher [18].

The biopsychosocial model of illness highlights the importance of open communication in clinical settings, not only to address biological symptoms but also the psychological and social challenges faced by patients [21]. Given that SRH can be impaired in men diagnosed with IA, and that it is considered an important contributor to their quality of life, the question remains: why is this topic rarely discussed between patients diagnosed with IA and their rheumatologists? Answering this question is challenging due to the subjective and sensitive nature of SRH issues. Factors such as fear of invading privacy and lack of confidence in addressing the topic often create barriers to open discussions about SRH in clinical settings. Furthermore, cultural factors are considered to be one of the most important subjective factors that influence sexual health across the world [22].

Our objective is to explore and describe the viewpoints of rheumatologists and male patients diagnosed with IA concerning the factors that influence the discussion about SRH with each other in a multi-cultural setting.

## Methods

### Q-methodology

In this study we use Q-methodology, which combines characteristics of qualitative and quantitative approaches for systematically exploring and explaining patterns in subjectivities (e.g., viewpoints, opinions, beliefs) around sensitive topics and identifying consensus and contrasts between them [23]. This method has been used to study views on complex subjective topics like organ donation [24], treatment adherence [25] and egg freezing [26]. Previously, we used this method to describe the impact of IA on male sexual health before and identified communication barriers between HCPs and patients as unmet needs that warrant further research [7, 27].

**Fig. 1:**
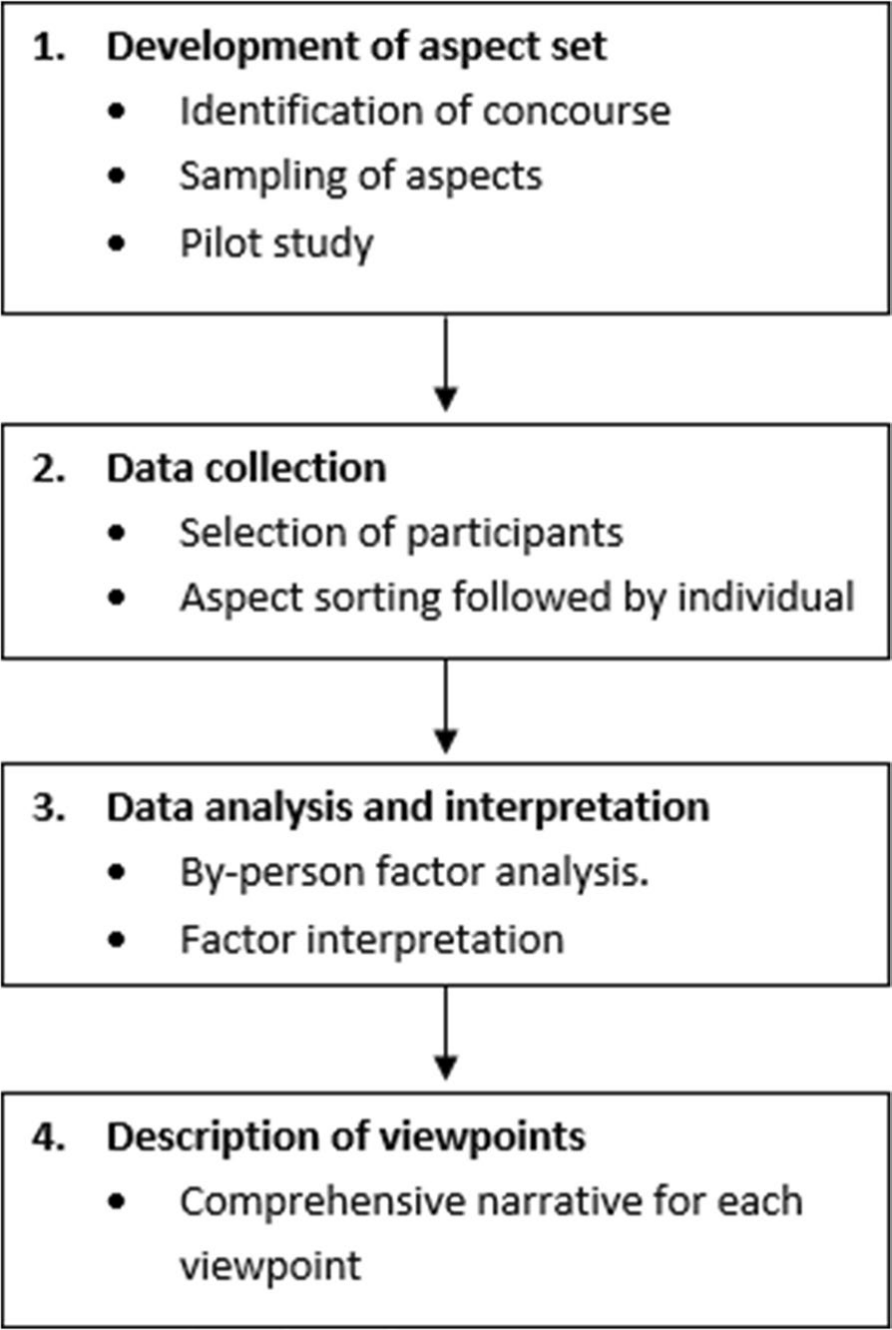
The stages of Q-methodology study

The whole process of a Q-methodology study can be summarized in four stages (see Fig. 1) [28]. Information on the use of Q-methodology in healthcare research can be found elsewhere [29]. Furthermore, a checklist on how to report a Q-methodology study is included as supplemental Table 1 [29].

### Development of aspect set

In the initial phase of the study design, two researchers (LFP, ER) collected candidate statements, i.e., aspects that influence the discussion of SRH between rheumatologists (and other HCPs) and male patients. This was based on a non-systematic review of scientific (PubMed), empirical, and popular literature (e.g., online forums, blogs) on this topic. This process resulted in an initial set of 68 aspects.

Furthermore, to attain a comprehensive list of potentially relevant aspects, 38 patients and 51 rheumatologists from The Netherlands (NL) and Mexico (MX) completed a questionnaire that included multiple ‘free text’ questions about this topic. Their responses contributed a total of 38 additional aspects from the experiences of the population that was going to be studied.

The total list of potential aspects was translated into Spanish and Dutch by professional translators with experience in translations for scientific publications. To evaluate the comprehensibility and comprehensiveness of this list, it was discussed with five rheumatologists and four patients (NL/MX). In addition, one expert in the field of Q-methodology (JvE) was consulted to provide methodological advice on the selection and formulation of aspects. Following these discussions, several adjustments were made: some aspects were excluded from the initial list because they covered similar topics, and the wording of several aspects was revised. At the end of this phase, a draft set of 34 aspects for rheumatologists and 32 aspects for patients, representative for the original long list, remained for pilot testing.

To further test the comprehensiveness and comprehensibility of these two sets of aspects and the other interview materials, a pilot study involving ten rheumatologists and four patients was conducted (NL/MX). Based on the results, no modifications to the materials, including the set of aspects, were deemed necessary. Therefore, the fourteen participants from the pilot study were retained for the main study. The sets of aspects used in the main study (in Dutch and Spanish) can be found in supplemental Table 2.

### Data collection

Participants were invited for an individual interview in their local hospital. Each session was moderated by LFP (bilingual, Spanish native speaker) and ER (bilingual, Dutch native speaker) and started with instructions for the study.

Firstly, participants were introduced to the concept of SRH using a PowerPoint presentation. This presentation included a definition of SRH that emphasized it is not solely about the physical act of sex but encompasses a broader understanding of sexual well-being. We encouraged participants to use their own interpretation of SRH in their responses. This approach aimed to ensure a comprehensive understanding of the term and its relevance to their personal experiences.

**Fig. 2:**
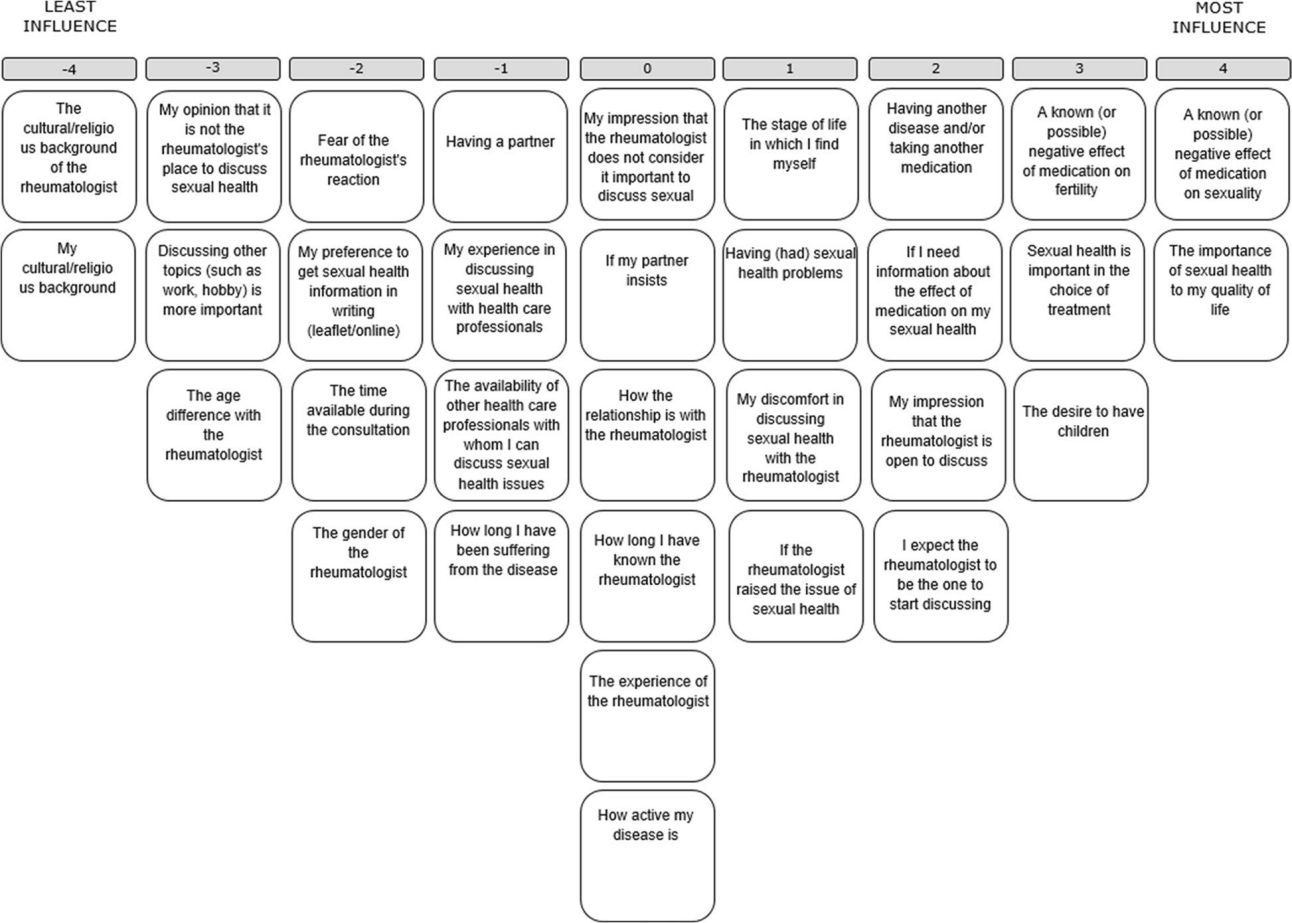
Sorting grid and aspects, example of final result. Participants first placed aspects which for them have an influence on discussing SRH on the right side of the score sheet. They placed the two aspects which have the most influence in the two spots in the extreme right column (+4), followed by the next three aspects which have the most influence (+3), and so on. In the same manner, respondents ranked aspects which for them have the least or no influence and those that they found to be neutral on the left side and in the center of the sorting grid, respectively, until all aspects were placed on the sorting grid with only one aspect placed in each cell. Participants were encouraged to review the final result and, if necessary, make any changes

Thereafter, participants were presented with the aspects printed on cards, in random order, and asked to carefully read all cards. They were asked to consider each aspect in relation to the question ‘What aspects influence the discussion of SRH with your rheumatologist / male patients diagnosed with IA?’ and to sort them into three piles representing aspects that had the most influence, the least influence and found to be neutral or irrelevant. The participants were then instructed to read the cards in each pile once again prior to ranking them on the sorting grid. (See Fig. 2). They started with the pile containing aspects that had ‘most influence’ according to themselves, followed by those in the pile ‘least influence’ pile and finally the neutral pile.

After ranking the aspects, participants were asked several open-ended questions. They were asked to explain the placement of certain aspects on the sorting grid and all participants elaborated on the two aspects that had the most and least influence according to them. Also, they were invited to discuss any aspect they found interesting or if there was an aspect that was not considered in the set. The interviews were voice recorded. The duration of this whole process, which included both the aspect ranking and interviews, was highly variable, ranging from 16 min to 97 min.

Finally, participants were asked to fill in a questionnaire, which included questions regarding their demographic characteristics and their medical history.

### Data analysis and interpretation

Individual aspect rankings were subject to factor extraction, followed by varimax rotation) using KADE 2.0.0 [30]. Solutions consisting of factors with Eigenvalue larger than one and at least two participants significantly associated (*p* < 0.05) were assessed and three-factor solutions were selected for both groups of participants after inspection of statistical information (i.e., explained variance and number of defining variables per factor) and the coherence and interpretability of the factors. These factors were interpreted as viewpoints on aspects that influence the discussion of SRH between rheumatologists and male patients diagnosed with IA. Interpretations were based on composite (i.e., weighted average) statement rankings for each factor and the qualitative materials of respondents associated with the factor collected during the interviews. In addition to the characterizing aspects for each factor (i.e., those ranked in the outer two columns of the grid for each of the viewpoints according to the composite sort for the factor), distinguishing aspects per factor (i.e., those whose rankings in one viewpoint differed significantly from those in the other viewpoints) and consensus aspects across factors (i.e., those whose rankings did not differ significantly between any pair of viewpoints) by-person factor analysis (i.e., centroid were identified.

### Description of viewpoints

The interpretation and description of each factor as a viewpoint was based on the ranking of the aspects and the qualitative data collected during the interviews from participants whose rankings were associated with that viewpoint (*p* < 0.05) explaining their ranking of the aspects. Description of the viewpoints involves developing narratives for each viewpoint based on the ranking of the aspects within a factor and relative to their ranking in other factors, also drawing on (and citing) the qualitative data of participants who fall under the viewpoint [29].

### Patient and public involvement

Six male patients diagnosed with IA (four were active members of the research advisory board from the Department of Rheumatology of the Erasmus University Medical Center, NL) and five rheumatologists (NL/MX) were involved in the design of the research question and the development of the statement set, the patient information leaflet and the invitation letter. In a pilot study, four patients and ten rheumatologists evaluated the statement set and the other interview materials. We also assessed the feasibility of the study in terms of the burden of the interview on participants.

### Participants

Participants were recruited between February 2022 and June 2023. Men with IA who are 18 years or older and rheumatologists who regularly treat male patients diagnosed with IA were invited. Participants had to be proficient in either Dutch or Spanish. As the aim of a Q-methodology study is to explore the variety of viewpoints that exist on a topic, not to make claims about the percentage of people holding them, participants were gathered purposively to ensure diversity. Therefore, recruiters were instructed to invite participants with different cultural and religious backgrounds as well as different health care/working environment settings (public vs. private sector). To promote diversity, four researchers (LFP, ER, AV and RD) frequently informed recruiters about the progress of inclusion of participants. Data collection in each country proceeded until saturation was achieved, which was considered to be attained when around 30 interviews per group consecutive interviews had revealed no significant new viewpoints as compared to earlier interviews.

### Ethics

This study was reviewed and approved by the Medical Ethics Committee of the Erasmus University Medical Center (MEC-2021-0385) and Instituto Nacional de Cardiologia Ignacio Chavez (NCAR-DG-DI-CI-EVAL-O63-2021). Written informed consent was obtained from all participants. All participants received financial compensation for their travel and parking costs and a gift card with a value of 20–30 euro.

## Results

120 participants were included (i.e., 60 patients and 60 rheumatologists). Demographic characteristics of the study population, are presented in Table 1.

**Table 1:** Demographic characteristics of participants

**Patients**			
	**All patients**	**The Netherlands**	**Mexico**
Participants, n (%)	60	30	30
Age, mean (SD)	44.7 (15.1)	44.1 (13.1)	45.4 (17.2)
Age at diagnosis, mean (SD)	33.8 (16.2)	33.0 (16.9)	34.6 (15.9)
Religious, n (%)	40 (66.0)	13 (46.5)	27 (93.1)
Disease duration years, mean (SD)	10.9 (10.2)	11.1 (10.1)	10.9 (10.6)
Diagnosis, n (%)			
- Rheumatoid Arthritis	24 (41.3)	13 (44.8)	11 (37.9)
- Spondyloarthropathy	15 (25.6)	6 (20.6)	9 (31.1)
- Psoriatic Arthritis	9 (15.5)	5 (17.2)	4 (13.8)
Currently in a relationship, n (%)	37 (64.9)	19 (67.8)	18 (62.1)
Number of children, mean (SD)	1.5 (1.5)	1.13 (1.3)	1.86 (1.70)
Active desire to have children, n (%)	13 (22.4)	9 (31.1)	4 (13.8)
Effect of IA on sexual health, n (%)	22 (37.9)	12 (41.9)	10 (34.5)
Erection problems, n (%)	20 (34.5)	9 (31.1)	11 (37.9)
**Rheumatologists**			
	**All rheumatologists**	**The Netherlands**	**Mexico**
Participants, n	60	30	30
**Age**,** n (%)**			
- < 25	- 0	- 0	- 0
- 25–34	- 12 (20.0)	- 3 (10.0)	- 9 (30.0)
- 35–44	- 24 (40.0)	- 13 (43.3)	- 11 (36.7)
- 45–54	- 15 (25.0)	- 11 (36.7)	- 4 (13.3)
- 55–64	- 7 (11.6)	- 3 (10)	- 4 (13.3)
- > 65	- 2 (3.3)	- 0	- 2 (6.7)
**Female**,** n (%)**	- 31 (51.7)	16 (53.3)	15 (50.0)
**Experience**,** n (%)**			
- In training	- 8 (13.3)	- 4 (13.3)	- 4 (13.3)
- < 5 years	- 13 (21.6)	- 7 (23.3)	- 6 (20.0)
- 5–15 years	- 23 (38.8)	- 13 (43.3)	- 10 (33.3)
- > 15 years	- 16 (26.6)	- 6 (20.0)	- 10 (33.3)
**Professional environment**,** n (%)**			
- University hospital	- 20 (33.3)	- 10 (33.3)	- 10 (33.3)
- General hospital	- 18 (30.0)	- 18 (60.0)	- 9 (30.0)
- Other/combination	- 22 (36.7)	- 2 (6.7)	- 11 (36.7)
**Religious**,** n (%)**	36 (60)	10 (33.3)	26 (86.6)

### Description of viewpoints – patients

The analysis revealed three viewpoints among patients diagnosed with IA. Fifty-two of the 60 patients were significantly associated with one of these viewpoints (*p* < 0.05). The viewpoints explained 44% of the variance in the ranking data and Table 2 shows the composite rankings of the aspects for each of the three viewpoints.

**Table 2:** Composite ranking of aspects for each viewpoint - patients

Statement		“Let’s talk about my wish to become a father”	“Let’s talk about sex”	“Let’s talk about my joints”
**1**	The gender of the rheumatologist	−3	−3	−4
**2**	The age difference with the rheumatologist	−3	−3	−4
**3**	The stage of life in which I find myself	0**	−1**	2**
**4**	The desire to have children	4**	−3**	−1**
**5**	Having a partner	1*	−2**	1*
**6**	My cultural/religious background	−4	−4	−2**
**7**	The cultural/religious background of the rheumatologist	−4*	−4*	−3*
**8**	The experience of the rheumatologist	−1	0	4**
**9**	How long I have known the rheumatologist	−2**	1	1
**10**	How the relationship is with the rheumatologist	0**	3	2
**11**	Having (had) sexual health problems	2**	−1	0
**12**	How active my disease is	2	2	4**
**13**	How long I have been suffering from the disease	0**	−2**	2**
**14**	Having another disease and/or taking another medication	0	0	3**
**15**	The time available during the consultation	−1	0**	0
**16**	Discussing other topics (such as work, hobby) is more important	−1	0**	−2
**17**	I expect the rheumatologist to be the one to start discussing the topic	0	0	0
**18**	If the rheumatologist raised the issue of sexual health during a previous consultation	1*	2*	−2**
**19**	My impression that the rheumatologist is open to discuss sexual health	1**	2**	0**
**20**	My impression that the rheumatologist does not consider it important to discuss sexual health	−2	1**	−2
**21**	Fear of the rheumatologist’s reaction	−3	−1**	−3
**22**	My discomfort in discussing sexual health with the rheumatologist	−2**	2**	−3**
**23**	My opinion that it is not the rheumatologist’s place to discuss sexual health	−2	−2	−1**
**24**	My experience in discussing sexual health with health care professionals	0	1	−1**
**25**	The availability of other health care professionals with whom I can discuss sexual health issues	1	1	0**
**26**	My preference to get sexual health information in writing (leaflet/online)	−1	−1	−1
**27**	If my partner insists	2**	−2**	1**
**28**	The importance of sexual health to my quality of life	3*	4	3
**29**	If I need information about the effect of medication on my sexual health	3	3*	2
**30**	Sexual health is important in the choice of treatment	2*	3**	1*
**31**	A known (or possible) negative effect of medication on fertility	4**	0	0
**32**	A known (or possible) negative effect of medication on sexuality	3	4*	3

Range -4 (least influence) to +4 (most influence)

*p <0.05, **p <0.01 versus all other factors

Separate analysis (data not shown) per country revealed the same trend towards three viewpoints with similar distinguishing and consensus statements. Therefore, the data was pooled and presented as a whole.

### Viewpoint 1: “Let’s talk about my wish to become a father”

Three characteristic aspects distinguish patients with this viewpoint. First, having a desire to have children (aspect 4: rank score + 4) and, consequently, a known (or potential) negative effect of medication on fertility (as. 31: +4) and sexuality (as. 32: +3, see Table 2) were considered as the most important aspects that influence discussing SRH with their rheumatologists; “*If the medication could impact my desire to have children*,* I want to discuss other alternatives*,* it is an obligation of the rheumatologist.*” Furthermore, they considered that sexual health is important to their quality of life (as. 28: +3), *”Sexual health is part of your life”*, and that it should be considered in the decision making process (as. 30: +2). Lastly, two additional aspects of influence were having the need for information on this regard (as. 29: +3) or that their partner insists (as. 27: +2); “*Fertility means having a baby… (pause)… and that is what scares me the most*,* to be less afraid I need more information on this*”.

Regarding communication issues, they were less likely to feel uncomfortable when discussing this topic with their rheumatologist (as. 22: −2); “*If you have a problem you have to discuss it and my discomfort has to be put aside*”. Furthermore, another aspect that facilitates discussing this topic is if patients have or have had problems related to sexual health (as. 11: +2); “*It is easier to talk about it if you already have experience with it”*.

With regards to the characteristics of their rheumatologist, two important aspects were of influence according to these patients; their impression that the rheumatologist is open to discuss the topic (as. 19: +1) and if the rheumatologist talked about this topic before (as. 18: +1). *“It is easier to talk about it because we already discussed sexual health”.* On the contrary, the rheumatologist’s age, gender, cultural or religious backgrounds and the kind of relationship they have with them were considered to have no influence (as. 2: −3, as. 1: −3, as. 7: −4, as. 10: 0). Furthermore, they were also open to discuss this topic with other HCPs such as a specialized nurse (as. 25: +1).

Viewpoint 1 had an eigenvalue of 16.62 and explained 28% of the variance. Eighteen participants (30%) were significantly associated with this viewpoint. Within the total sample of patients recruited for the study, patients statistically significantly associated with this viewpoint were younger (27.4 years), and a higher proportion had an active wish to become a father (38.9%) and to express that the IA had/has an effect on their family planning (44.4%).

### Viewpoint 2: “Let’s talk about sex”

SRH is important to their quality of life and should be considered during the decision-making process (as. 28: +4 and as. 30: +3); “*A side effect of medication that can negatively affect sexuality can also directly or indirectly impact your relationship*”. A significant difference compared to viewpoint 1 is that patients with this viewpoint were more likely to be influenced by a known or potential negative effect of their disease or treatment on their sexuality (as. 32: +4) than on their fertility (as. 31: 0) “*You don’t have to have an active desire to have children to be able to enjoy sex*”. This can be explained by the fact that having an active desire to have children was not relevant for them (as. 4: −3).

Regarding communication, patients with this viewpoint might feel motivated to start the conversation if they need information regarding the effect of medication on their sexuality (as. 29: +3). Nonetheless, this is mostly not a straightforward action as they were more likely to feel “uncomfortable” discussing this topic with their rheumatologists (as. 22: +2); “*I have had sexual health problems that might be related to my medication*,* but I didn’t dare to bring it up with my rheumatologist*”. On the contrary, some aspects can facilitate the conversation, such as having a good relationship with their rheumatologist (as. 10: +3); “*If the relationship is good*,* it does not matter (the uncomfortable feeling*)”. Also, having the impression that the rheumatologist value this topic as important (as. 20: +1) and is open to discuss the topic (as. 19:+2); “*If you are a sensitive person it is relatively easy to get the feeling that someone is open to discuss this topic*” or “*When he asked me about my sexuality I got the feeling that he was not comfortable talking about this topic with me*”.

Viewpoint 2 had an eigenvalue of 4.14 and explained 7% of the variance. Seventeen participants (28.3%) were significantly associated with this viewpoint. A higher proportion of the patients defining this viewpoint were single (53.3%) or had no active wish for having children (81.3%).

### Viewpoint 3: “Let’s talk about my joints”

Patients with this viewpoint considered that having a discussion about SRH depends almost exclusively on how active the disease is (as. 12: +4); “*Before I came to the rheumatologist my quality of life was super bad*,* I couldn’t do anything*,* thinking about sex is then impossible*” or “*I use my medication to feel better and be able to care for my children even though the medication can negatively affect some things*”. Being diagnosed with another disease or using other medication (as. 14:+3) was also an important aspect of influence.

Contrary to the other viewpoints, some aspects related to the relation with their rheumatologist were seen to have influence on discussing SRH. Having an experienced rheumatologist (as. 8: +4), having a good relationship with him/her (as. 10: +2) and having known each other for some time (as. 13: +2) came forward as aspects that facilitate the discussion. “*The rheumatologist knows what is important for me*,* I trust him to inform me about important issues*”. Furthermore, “*The good relationship I have with my rheumatologist makes having a discussion about sex easier*”.

On a personal level, patients with this viewpoint were more likely to be influenced by the phase of the life they are currently in (as. 3:+2) and by their partners (as. 5: +1; as. 27: +1); “*I am 66 years old but fortunately I still have a good time with my partner*” or “*Not everybody has a partner and sometimes you need a partner to stimulate you to get the help you need*”. A desire to have children (as. 4:−1) and a known negative (or potential) negative effect on fertility (as. 31:0) were not relevant in this viewpoint; “*That would have been an interesting conversation to have… 30 years ago*,* but it never happened. This should be discussed with all young patients*,* it is so important*,* I really regret that I never asked about this before*”.

Receiving information regarding known (or potential) negative effects on sexuality (as. 32:+3) was considered important as sexual health is valued for their quality of life (as. 28: +3); “*I would really appreciate if my rheumatologist informs me about potential sexuality side effects*,* then I would really have to think about it*,* this is information I really want to have*”.

Regarding communication, patients with this viewpoint were less likely to feel discomfort (as. 22, −3) when discussing SRH.

Viewpoint 3 had an eigenvalue of 5.25 and explained 9% of the variance. Seventeen participants (28.3%) were significantly associated with this viewpoint. Within the total group of included patients, these men were older (53.2 years), diagnosed at an older age (40.2 years, thus after reproductive age), more likely religious (81.2%), and had more children (2.6) and more erection problems (50%).

### Consensus aspects

Across viewpoints there was agreement that the gender of the rheumatologist (as. 1: −3, −4, −3) and the age difference with the rheumatologist (as. 2: −3, −4, −3) had little influence on discussing SRH, and that this is a topic that can be discussed with the rheumatologist (as. 23: −2, −1, −2).

### Description of viewpoints - rheumatologists

The analysis revealed three viewpoints among rheumatologists in NL and MX. Forty-seven of the 60 rheumatologists were significantly associated with one of these viewpoints (*p* < 0.05). The viewpoints explained 55% of the variance in the ranking data and Table 3 shows the composite rankings of the aspects for each of the three viewpoints together with the distinguishing and consensus aspects.

**Table 3:** Composite ranking of aspects for each viewpoint - rheumatologists

Statement		“Let’s talk about side effects”	“Let’s talk about your desire to have children ”	“Let’s talk about your joints”
**1**	The age difference with the patient	−1**	−3*	−4*
**2**	The patient’s stage in life	1	1	3**
**3**	The patient’s desire to have children	3	4	3
**4**	Whether the patient has a partner	−1**	1	1
**5**	My cultural/religious background	−4	−4	−4
**6**	The patient’s cultural/religious background	−1	−1	1**
**7**	The patient’s socioeconomic level/education level	−3**	0**	0**
**8**	How long I have known the patient	−1*	−2*	0*
**9**	How the relationship is with the patient	1	0**	2
**10**	Personal experiences with sexual health	−3	−1*	−2
**11**	The patient’s sexual health issues	2	2	2
**12**	How active the disease is	−2**	1**	4**
**13**	The duration of the disease	−3**	1	0
**14**	Comorbidity and/or medication	−2**	2	1
**15**	The time available during the consultation	2	−1**	3
**16**	Discussing other topics (such as work, hobby) is more important	−1**	−2	−2
**17**	I expect the patient to be the one to start discussing the topic	0	−3**	−1
**18**	If the patient raised the issue of sexual health during a previous consultation	4**	1	1
**19**	My impression that the patient is open to discuss sexual health	3**	0	0
**20**	My impression that the patient does not consider it important to discuss sexual health	1**	−2	−2
**21**	My fear of violating the patient’s privacy	0**	−1*	−3*
**22**	My discomfort in discussing sexual health with patients	0**	−3	−3
**23**	My opinion that it is not the rheumatologist’s place to discuss sexual health	−4	−4	−3
**24**	My experience in discussing sexual health with patients	0	0	−1
**25**	My ability to engage in sexual health conversations	0	0	−1
**26**	The availability of other health care professionals with whom the patient can discuss sexual health	0	−1	−1
**27**	My preference to provide sexual health information in writing (leaflet/online)	−2	−2	−2
**28**	The importance of sexual health to the patient’s quality of life	2**	3	4
**29**	The available information on the effect of the medication on sexual health	2	2	0**
**30**	A known (or possible) negative effect of the medication on fertility	4	4	2**
**31**	A known (or possible) negative effect of the medication on sexuality	3	3	2**
**32**	Sexual health is important in the choice of treatment	1**	3**	−1**
**33**	The effect of the medication on sexual health is important for adherence to treatment	1	2**	1
**34**	My interest in the topic of sexual health	−2**	0	0

Range -4 (least influence) to +4 (most influence)

*p <0.05, **p <0.01versus all other factors

Separate analysis (data not shown) per country revealed the same trend towards three viewpoints with similar distinguishing and consensus statements. Therefore, the data was pooled and presented as a whole.

### Viewpoint 1: “Let’s talk about side effects”

The known or potential negative side effects of medication on fertility (as. 30: +4) and sexual health (as. 31: +3) were the most influential aspects that trigger rheumatologists with this viewpoint to discuss male SRH (See Table 3). They feel responsible for informing their patients about these side effects, especially their young patients, and more specifically those with an active wish to become a father (as. 3: +3); “*I often talk about this topic with my young patients*,* specifically when I start new medication that I know that may cause fertility or sexuality side effects*”.

To discuss this topic, an important distinguishing characteristic of this viewpoint is that patient-related aspects can be considered as “conditioning aspects”. The most important being the fact that a patient approached this topic earlier (as. 18: +4); ”*The patient has to come to me with a very specific question about this topic*”.

Furthermore, this viewpoint is highly influenced by the impressions of rheumatologists that the patient does not consider discussing sexual health as important (as. 20: +1) or that they are (not) open to discuss the topic (as. 19: +3) “*Sometimes I feel that the patient is a bit restless about something but does not dare to say it. If you ask them “is there anything else you want to discuss with me?” …it is almost always something about sexuality*”. Limited time during consultations (as. 15: +2) and considering that other topics might be more relevant (as. 16: −1) were frequently mentioned as limiting aspects to address this topic with their patients.

These conditions and limitations can be partially explained by the fact that these rheumatologists with this viewpoint were more likely to feel discomfort (as. 22: 0) or fear of invading the patient’s privacy when discussing this topic (as. 21: 0); “*It is not a topic I am looking forward to discuss with my patients*”.

During the interviews it became evident that rheumatologists with this viewpoint don’t really discuss this topic with their patients but rather “inform” them about the side effects. In this regard, they are more likely to have less interest in this topic (as. 34: −2), they give less relevance to the importance of sexual health to quality of life (as. 28: +2) and were less likely to consider sexual health as an important aspect during the decision-making process (as. 28: +2); “*It does not matter if I like to talk about it or not*,* but a few times you have to talk about it with your patients*”.

Viewpoint 1 had an eigenvalue of 25.6 and explained 43% of the variance. Twenty-six participants (43.3%) were significantly associated with this viewpoint. Within the recruited population of rheumatologists, those statistically significantly associated with this viewpoint more often worked in an academic hospital (42.3%).

### Viewpoint 2: “Let’s talk about your desire to have children”

Having an active wish to become a father (as. 3: +4) was the “automatic” trigger to discuss male SRH for rheumatologists with this viewpoint; “*I ask all my patients between 18–40 years if they have an active desire to have children*”.

Subsequently, they feel obligated to inform their patients about known or potential negative side effects of medication on fertility (as. 30: +4) and in lesser degree, on sexuality (as. 31: +3); “*If you intervene with medication that can affect fertility you have the responsibility to provide your patients with all the available information*”.

Rheumatologists with this viewpoint also believe that patients’ desire to have children has a profound effect on the therapeutic decision-making process (as. 32: +3) and therapy compliance (as. 33: +2). “*A patient asked me once; you are prescribing me a new medication*,* but my question is how would this affect what I really want (having a baby)?”*. Furthermore, they highly value the importance of sexual health on their patients’ quality of life (as. 28:+3).

Independent from the desire to have children, disease activity (as. 12: +1) is also a frequent triggering aspect that “opens the door” for discussing SRH. This is related to the fact that rheumatologists might adjust treatment and this in turn leads to prescribing new medication with potential side effects; “*If the disease is active and I need to start new medication with potential side effects on fertility*,* I will tell them about it*”.

Furthermore, they don’t feel discomfort (as. 22 − 3) or fear of invading patient’s privacy (as. 21: −1) when discussing SRH, or limited by the available time for consultations (as. 15: −1). In short, they are more likely to spontaneously start these kinds of conversations with patients *with an active wish to become a father*.

Viewpoint 2 had an eigenvalue of 4.07 and explained 7% of the variance. Thirteen participants (21.6%) were significantly associated with this viewpoint. Within the recruited population of rheumatologists, those statistically significantly associated with this viewpoint more often were female rheumatologists (83%).

### Viewpoint 3: “Let’s talk about your joints”

Although they considered sexual health as an important contributor to their patient’s quality of life (as. 28: +4), controlling the disease activity was the most important aspect for rheumatologists with this viewpoint, essentially dictating when to discuss reproductive and sexual health with their male patients (as. 12: +4); “*I am a rheumatologist*,* I am there for the patient and the disease has to be treated*,* otherwise they will have all kind of problems*,* including fertility problems*” or “*First treat the disease*,* then the rest…”.*

They rarely discuss this topic with their patients, with the exemption of men with an active wish to become a father (as. 3: +3) or young men (as. 2: +3) “*I don’t think of this automatically*,* there must be a trigger that is initiated by the patient (e.g. patient informing them of their active desire to have children)*”. They acknowledge that time was also a limiting aspect to approach this topic (as. 15: +3).

Furthermore, known or potential side effects of medication on fertility or sexuality were not considered an automatic trigger to initiate the discussion (as. 30:+2 and as. 31: +2); “*Sometimes I decide not to mention a lot of “potential” side effects because I am afraid that the patient will get scared and won’t take his medication*”.

One distinguishing characteristic of this viewpoint is that demographic characteristics of their patients were considered more influential, in particular the religious, cultural and socioeconomic background of patients (as. 6; 1 and as. 7;0); “*Why should I talk about this topic with some of my patients*,* they would probably do not understand what I say*” or “*Maybe with some of these patients (low socioeconomic status) we do not talk about it*”.

Rheumatologists with this viewpoint were not afraid they will be invading their patient’s privacy or don’t feel uncomfortable when discussing this topic with them (as. 21; −3, as. 22; −3). During the interviews it became evident that this applied when focusing the discussion on fertility, not necessarily on sexual health.

Viewpoint 3 had an eigenvalue of 5.25 and explained 9% of the variance. Seventeen participants (28.3%) were significantly associated with this viewpoint. Within the total group of included rheumatologists, this group was more likely to work in non-academic hospitals (100%) and be a male rheumatologist (77.8%).

### Consensus aspects

Participating rheumatologists largely disagreed that it is not their place to discuss SRH with their patients (as. 23: −4, −3, −4). They agreed that an active wish to become a father (as. 3: +3, + 4, +3) was an influential aspect to discuss SRH with their male patients, and that their own cultural and religious background (as. 5: −4, −4, −4) had no influence on discussing this topic.; *“I am a professional*,* my personal background should not influence how I treat my patients*”.

## Discussion

This study describes viewpoints on the aspects that influence discussing SRH between rheumatologists and male patients with IA in two countries with distinct cultures and healthcare systems. Three viewpoints were identified and described per group, with no major differences between countries observed. Rheumatologists are mostly influenced by patients having an active wish to become a father and discussing potential side effects of medication (fertility > sexuality), while patients are influenced by a much more diverse pool of aspects. In other words, when raising this topic, rheumatologists mostly focus on fertility and reproduction, while patients’ needs and interests around this topic can be much broader.

This distinction may also reflect a fundamental divide between traditions in medical science, where biological science (focusing on biology and physiology) contrasts with biosocial medicine (full integration of the person’s biology and human behavior) [31].The potential mismatch in viewpoints between rheumatologists and patients on the aspects that trigger them to discuss SRH can help to explain why this topic remains a “neglected” and perhaps also somewhat controversial topic in Rheumatology.

Several “historical” factors may partially explain this mismatch between rheumatologists and patients. The lack of formal sexual health curricula in medical schools [32, 33] and the predominance of research focused on potential side effects of medication on fertility [19, 34–36] may have contributed to the stronger focus of rheumatologists on aspects related to male fertility. This reflects a broader trend where medical practice often emphasizes biological science over the psychosocial dimensions of health. Another potential mismatch is that both parties may expect the other to initiate the discussion about SRH, resulting in silence about this important topic [37]. This phenomenon can be understood through the concept of a “two-way taboo” where both patients and clinicians avoid initiating discussions about sensitive topics, reinforcing the silence around SRH [38–40].

The rheumatologist’s “automatic” trigger to discuss SRH is the patient’s active wish to become a father. Nonetheless, this is only relevant for a specific group of mainly young patients. In addition, young patients might require information on SRH, including family planning, years before their wish to conceive becomes active. Lastly, this study shows that patients want to discuss SRH with their rheumatologists, also when their family planning is fulfilled. Therefore, rheumatologists are encouraged to approach this topic early, often and in a proactive way.

Regarding “proactively” initiating conversations about SRH, there is ongoing debate about whether it is primarily the HCP responsibility to start this discussion [41]. Our findings support the need for rheumatologists to take a proactive role, but they also highlight the importance of creating an environment where patients feel comfortable initiating these discussions if they choose to.

Independent of the active desire to become a father, rheumatologists and patients agree that discussing known or potential side effects of medication on fertility and sexuality is important. However, in this regard rheumatologists are more comfortable talking about potential side effects related to fertility and reproduction (e.g. sperm quality or testosterone levels), than about side effects related to sexuality (e.g. ED). On the other side, patients, especially those without an active wish to become a father, are more interested in discussing side effects related to sexuality.

Modern Rheumatology is characterized by being a patient-centered specialty where patients are actively involved in the decision making process. It is known that patients need to be confident and well-informed about their care to be fully engaged with their care [42]. In this regard, the American College of Rheumatology recommends discussing SRH with patients “early and often” but lack specific recommendations on how to succeed in this [10].

To facilitate the “early and often” discussion of SRH, we encourage rheumatologists to inform their patients early in the course of their disease that the disease itself or medication can impact their SRH. Acknowledging this association should be considered as one of the most important steps to efficiently approach this topic; not only do patients become aware of this association, but more importantly, rheumatologists let the patient know that they are open to discuss SRH issues. Consequently, when patients experience SRH problems or have questions regarding this matter, these two important actions (“inform and acknowledge”) may facilitate the conversation in the outpatient clinic.

During follow-up consultations, the use of other facilitators such as specific pre-consultation questionnaires that include SRH questions and the involvement of other HCPs such as specialized nurses may facilitate the discussion of SRH problems [43, 44]. The implementation of this approach in our Reproductive Rheumatology outpatient clinic has resulted in a high patient satisfaction rates and improved clinical outcomes [45].

The qualitative data obtained during the interviews exposed a significant “hidden” aspect of influence for some rheumatologist when discussing (or not) SRH; the assumptions they make about the patient. This is a known form of bias (implicit bias), defined as “a negative attitude, of which one is not consciously aware, against a specific social group” [46]. Regarding male SRH, rheumatologists often assumed that older patients (> 55 years) or patients with specific religious or cultural backgrounds may have no interest in discussing sexuality. Addressing this implicit bias requires targeted training and awareness programs for HCPs to ensure they do not make assumptions based on age, religion, or cultural background, which can hinder open communication [47].

Our study has several strengths. By combining qualitative and quantitative data using Q-methodology we were able to describe how the same topic can be very differently perceived by two parties. On the one side, the study describes how in patients, very personal aspects influence the discussion of this sensitive topic (having a wish to become a father, having (or not) a partner, having ED symptoms). On the other side, it describes how for rheumatologists, the topic is more “legal” or “corporate” (e.g. feeling obligated to discuss side effects of medications). Furthermore, the study describes multiple characteristics that could have been easily missed by conventional questionnaires and that were crucial to understand the described viewpoints.

Moreover, this study was conducted in two countries that have very different cultural backgrounds and health-care systems. In general, the aspects triggering the discussion of SRH were similar in both countries. Lastly, although a Q-methodology study is not designed to answer epidemiological questions our samples are quite large (*n* = 60 and *n* = 60) for a Q-methodology study.

An important limitation of this study is that its results are not generalizable to different populations. Another limitation is that because the Q-sets were slightly different for both groups (patients and rheumatologists) no direct comparisons between both groups can be made. Furthermore, since there was no know relation between the participating patients and rheumatologists, nothing can be said about ‘match in patient-doctor’ communication’. Lastly, data on participants’ sexual preferences and ethnicity were not systematically collected, which could influence the reasons participants might want to discuss SRH and their comfort level in doing so.

For future research, important research recommendations can be made. First, epidemiological research is needed to establish the prevalence of these viewpoints in the general population. Second, studies evaluating the impact of educating HCPs regarding communication in sexual health are encouraged. The results of these future studies can be used to design evidence-based clinical pathways that can be implemented in daily practice [48]. Altogether, it can be expected that these actions result in an much needed new approach of this currently neglected topic in Rheumatology.

In conclusion, our study describes the different viewpoints on the aspects that influence discussing SRH between rheumatologists and male patients with IA. Rheumatologists are more likely to initiate the discussion about this topic if their patients are young and have an active wish to have children. On the other hand, patients are influenced by more aspects that go beyond reproduction. Patients should be informed about the potential impact of SRH of IA early during the course of their disease and provided with “facilitators” to discuss this topic throughout the course of their disease.

## Supplementary Information

Supplementary Material 1.

Supplementary Material 2.

## Data Availability

The data that support the findings of this study are available from department of Rheumatology Erasmus Medical Center, but restrictions apply to the availability of these data, which were used under license for the current study, and so are not publicly available. Data are however available from the authors upon reasonable request and with permission of department of Rheumatology Erasmus Medical Center.

## Abbreviations

ED: Erectile dysfunction
HCPs: Health care professionals
IA: Inflammatory arthritis
NL: Netherlands
MX: Mexico
SD: Standard deviation
SRH: Sexual and reproductive health
