# Supplementary material for: Discussing male sexual and reproductive health in the rheumatology outpatient clinic: a Q-methodology study
Source: BMC Rheumatol. 2024 Dec 5;8:67. doi: 10.1186/s41927-024-00441-3 (PMC11622486; doi:10.1186/s41927-024-00441-3)
Supplement: Supplementary file 1 — Supplementary Material 1. [file 41927_2024_441_MOESM1_ESM.docx]

***Supplemental Table 1.*** *Checklist to include when reporting a Q-methodological study by Churruca K et al (12) is licensed under* [*CC BY 4.0*](https://creativecommons.org/licenses/by/4.0/)*.*

| Checklist |
| --- |
| \| How items/statements for the Q-set were collected \| 🗹 \| \| --- \| --- \| \| How the statements were refined and reduced to produce the draft and final Q-set \| 🗹 \| \| The number of statements in the final Q-set \| 🗹 \| \| What, if any, piloting was done and what the results were \| 🗹 \| \| The materials used for the Q-sorting task including the ranking scale and anchors \| 🗹 \| \| How the Q-sorting task was administered \| 🗹 \| \| What, if any, other methods were used in conjunction with Q-sorting, and how the data captured by these methods was used in relation to Q-data \| 🗹 \| \| The techniques used for factor extraction and rotation \| 🗹 \| \| The software programs used to administer and/or analyse the data \| 🗹 \| \| The information used to decide the number of factors to extract, rotate and interpret \| 🗹 \| \| The amount of variance explained by the factor solution \| 🗹 \| \| The processes for interpreting the factors \| 🗹 \| \| A rich narrative for each factor that explains the shared meaning it represents, supported by Q-set statements, and participant quotes where available \| 🗹 \| |
