# Supplementary material for: Discussing male sexual and reproductive health in the rheumatology outpatient clinic: a Q-methodology study
Source: BMC Rheumatol. 2024 Dec 5;8:67. doi: 10.1186/s41927-024-00441-3 (PMC11622486; doi:10.1186/s41927-024-00441-3)
Supplement: Supplementary file 2 — Supplementary Material 2. [file 41927_2024_441_MOESM2_ESM.docx]

**Supplemental table 2**. Original Statements (Dutch and Spanish)

**2a. Patients.**

| \| **Dutch** \| **Spanish** \| \| --- \| --- \| \| **Welke aspecten hebben invloed op het bespreken van uw seksuele gezondheid met uw reumatoloog?** \| **¿Qué aspectos influyen al hablar acerca de la salud sexual con el reumatólog@?** \| \| Het geslacht van de reumatoloog \| El género del reumatólog@ \| \| Het leeftijdsverschil met de reumatoloog \| La diferencia de edad con el reumatólog@ \| \| De levensfase waarin ik mij bevind \| La etapa de la vida en la que me encuentro \| \| Het hebben van een kinderwens \| El deseo de tener hijos \| \| Het hebben van een partner \| El hecho de tener pareja \| \| Mijn culturele/religieuze achtergrond \| Mi cultura/religión \| \| De culturele/religieuze achtergrond van de reumatoloog \| La cultura/religión del reumatólog@ \| \| De ervaring van de reumatoloog \| La experiencia del reumatólog@ \| \| Hoe lang ik de reumatoloog ken \| Cuánto tiempo hace que conozco al reumatólog@ \| \| Hoe de relatie is met de reumatoloog \| Cómo es la relación que tengo con el reumatólog@ \| \| Het hebben (gehad) van problemen op het gebied van seksuele gezondheid \| El hecho de tener (haber tenido) problemas de salud sexual \| \| Hoe actief mijn ziekte is \| Qué tan activa está mi enfermedad \| \| Hoe lang ik de ziekte heb \| Cuánto tiempo llevo padeciendo la enfermedad \| \| Het hebben van een andere ziekte en/of gebruik van andere medicatie \| El hecho de padecer otra enfermedad y/o de tomar otro medicamento \| \| De beschikbare tijd tijdens het consult \| El tiempo disponible durante la consulta \| \| Het bespreken van andere onderwerpen (zoals werk, hobby) is belangrijker \| Hablar sobre otros temas (como el trabajo, las aficiones) es más importante \| \| Ik verwacht dat de reumatoloog erover begint \| Espero que sea el reumatólog@ quien empiece a hablar sobre el tema \| \| Of de reumatoloog tijdens een eerder consult naar mijn seksuele gezondheid heeft geïnformeerd \| Si el reumatólog@ me preguntó sobre mi salud sexual en una consulta anterior \| \| Mijn indruk dat de reumatoloog open staat voor het bespreken van seksuele gezondheid \| Mi impresión de que el reumatólog@ está abierto a hablar sobre la salud sexual \| \| Mijn indruk dat de reumatoloog het bespreken van seksuele gezondheid niet belangrijk vindt \| Mi impresión de que el reumatólog@ no considera importante hablar de la salud sexual \| \| Angst voor de reactie van de reumatoloog \| El temor ante la reacción del reumatólog@ \| \| Ongemak om over seksuele gezondheid te praten met de reumatoloog \| La incomodidad que me produce hablar sobre la salud sexual con el reumatólog@ \| \| Mijn mening dat het bespreken van seksuele gezondheid niet thuis hoort bij de reumatoloog \| La opinión que tengo de que no es competencia del reumatólog@ hablar sobre la salud sexual \| \| Mijn ervaring met het bespreken van seksuele gezondheid met zorgverleners \| Mi experiencia abordando el tema de la salud sexual con los profesionales de la salud \| \| De beschikbaarheid van andere zorgverleners waarmee ik seksuele gezondheid kan bespreken \| La disponibilidad de otros profesionales de la salud con los que puedo hablar sobre la salud sexual \| \| Mijn voorkeur om informatie over seksuele gezondheid schriftelijk te krijgen (folder/online) \| El hecho de que prefiero recibir información sobre la salud sexual por escrito (folleto/en línea) \| \| Als mijn partner erop aandringt \| Si mi pareja insiste \| \| Het belang van seksuele gezondheid voor mijn kwaliteit van leven \| La importancia de la salud sexual para mi calidad de vida \| \| Als ik behoefte heb aan informatie over het effect van medicatie op mijn seksuele gezondheid \| Si necesito información sobre el efecto del medicamento en mi salud sexual \| \| Seksuele gezondheid is van belang bij de keuze van de behandeling \| La salud sexual es importante para la elección del tratamiento \| \| Een bekend (of mogelijk) negatief effect van de medicatie op de vruchtbaarheid \| Un efecto negativo conocido (o posible) del medicamento en la fertilidad \| \| Een bekend (of mogelijk) negatief effect van de medicatie op de seksualiteit \| Un efecto negativo conocido (o posible) del medicamento en la sexualidad \| \| ** Translation: Bureau voor Spaanstalige Dienstverlening* \| \| |
| --- | --- | --- | --- | --- | --- | --- | --- | --- | --- | --- | --- | --- | --- | --- | --- | --- | --- | --- | --- | --- | --- | --- | --- | --- | --- | --- | --- | --- | --- | --- | --- | --- | --- | --- | --- | --- | --- | --- | --- | --- | --- | --- | --- | --- | --- | --- | --- | --- | --- | --- | --- | --- | --- | --- | --- | --- | --- | --- | --- | --- | --- | --- | --- | --- | --- | --- | --- | --- | --- | --- |
|  |
|  |

**2b. Rheumatologists**

| \| **Dutch** \| **Spanish** \| \| --- \| --- \| \| **Welke aspecten hebben invloed op het bespreken van uw seksuele gezondheid met uw reumatoloog?** \| **¿Qué aspectos influyen al hablar acerca de la salud sexual con el reumatólog@?** \| \| Het geslacht van de reumatoloog \| El género del reumatólog@ \| \| Het leeftijdsverschil met de reumatoloog \| La diferencia de edad con el reumatólog@ \| \| De levensfase waarin ik mij bevind \| La etapa de la vida en la que me encuentro \| \| Het hebben van een kinderwens \| El deseo de tener hijos \| \| Het hebben van een partner \| El hecho de tener pareja \| \| Mijn culturele/religieuze achtergrond \| Mi cultura/religión \| \| De culturele/religieuze achtergrond van de reumatoloog \| La cultura/religión del reumatólog@ \| \| De ervaring van de reumatoloog \| La experiencia del reumatólog@ \| \| Hoe lang ik de reumatoloog ken \| Cuánto tiempo hace que conozco al reumatólog@ \| \| Hoe de relatie is met de reumatoloog \| Cómo es la relación que tengo con el reumatólog@ \| \| Het hebben (gehad) van problemen op het gebied van seksuele gezondheid \| El hecho de tener (haber tenido) problemas de salud sexual \| \| Hoe actief mijn ziekte is \| Qué tan activa está mi enfermedad \| \| Hoe lang ik de ziekte heb \| Cuánto tiempo llevo padeciendo la enfermedad \| \| Het hebben van een andere ziekte en/of gebruik van andere medicatie \| El hecho de padecer otra enfermedad y/o de tomar otro medicamento \| \| De beschikbare tijd tijdens het consult \| El tiempo disponible durante la consulta \| \| Het bespreken van andere onderwerpen (zoals werk, hobby) is belangrijker \| Hablar sobre otros temas (como el trabajo, las aficiones) es más importante \| \| Ik verwacht dat de reumatoloog erover begint \| Espero que sea el reumatólog@ quien empiece a hablar sobre el tema \| \| Of de reumatoloog tijdens een eerder consult naar mijn seksuele gezondheid heeft geïnformeerd \| Si el reumatólog@ me preguntó sobre mi salud sexual en una consulta anterior \| \| Mijn indruk dat de reumatoloog open staat voor het bespreken van seksuele gezondheid \| Mi impresión de que el reumatólog@ está abierto a hablar sobre la salud sexual \| \| Mijn indruk dat de reumatoloog het bespreken van seksuele gezondheid niet belangrijk vindt \| Mi impresión de que el reumatólog@ no considera importante hablar de la salud sexual \| \| Angst voor de reactie van de reumatoloog \| El temor ante la reacción del reumatólog@ \| \| Ongemak om over seksuele gezondheid te praten met de reumatoloog \| La incomodidad que me produce hablar sobre la salud sexual con el reumatólog@ \| \| Mijn mening dat het bespreken van seksuele gezondheid niet thuis hoort bij de reumatoloog \| La opinión que tengo de que no es competencia del reumatólog@ hablar sobre la salud sexual \| \| Mijn ervaring met het bespreken van seksuele gezondheid met zorgverleners \| Mi experiencia abordando el tema de la salud sexual con los profesionales de la salud \| \| De beschikbaarheid van andere zorgverleners waarmee ik seksuele gezondheid kan bespreken \| La disponibilidad de otros profesionales de la salud con los que puedo hablar sobre la salud sexual \| \| Mijn voorkeur om informatie over seksuele gezondheid schriftelijk te krijgen (folder/online) \| El hecho de que prefiero recibir información sobre la salud sexual por escrito (folleto/en línea) \| \| Als mijn partner erop aandringt \| Si mi pareja insiste \| \| Het belang van seksuele gezondheid voor mijn kwaliteit van leven \| La importancia de la salud sexual para mi calidad de vida \| \| Als ik behoefte heb aan informatie over het effect van medicatie op mijn seksuele gezondheid \| Si necesito información sobre el efecto del medicamento en mi salud sexual \| \| Seksuele gezondheid is van belang bij de keuze van de behandeling \| La salud sexual es importante para la elección del tratamiento \| \| Een bekend (of mogelijk) negatief effect van de medicatie op de vruchtbaarheid \| Un efecto negativo conocido (o posible) del medicamento en la fertilidad \| \| Een bekend (of mogelijk) negatief effect van de medicatie op de seksualiteit \| Un efecto negativo conocido (o posible) del medicamento en la sexualidad \| |
| --- | --- | --- | --- | --- | --- | --- | --- | --- | --- | --- | --- | --- | --- | --- | --- | --- | --- | --- | --- | --- | --- | --- | --- | --- | --- | --- | --- | --- | --- | --- | --- | --- | --- | --- | --- | --- | --- | --- | --- | --- | --- | --- | --- | --- | --- | --- | --- | --- | --- | --- | --- | --- | --- | --- | --- | --- | --- | --- | --- | --- | --- | --- | --- | --- | --- | --- | --- | --- |
| ** Translation: Bureau voor Spaanstalige Dienstverlening* |
